# Supplementary material for: Most Random-Encounter-Model Density Estimates in Camera-Based Predator–Prey Studies Are Unreliable
Source: Animals (Basel). 2024 Nov 22;14(23):3361. doi: 10.3390/ani14233361 (PMC11639839; doi:10.3390/ani14233361)
Supplement: Supplementary file 1 [file animals-14-03361-s001.zip › Table S1.pdf]

Table S1. Model selection results for all fitted generalized linear models used to estimate trends in predator-prey REM studies and predator-prey REM density estimates. Models were fitted to each response variable (number of studies or number of density estimates) that followed Poisson or negative-binomial distributions and estimates were produced from the most supported models based on Akaike's Information Criterion corrected for small sample size (AIC_c).

**Models**	**df**[a]	**LL**[b]	**AIC_c**	**ΔAIC_c**[c]
No. Studies Across Time				
Poisson(Studies) ~ $\beta_0 + \beta_1$(Year)	2	-14.05	33.00	0.00
Neg-Binomial(Studies) ~ $\beta_0 + \beta_1$(Year)	3	Did not converge		
No. Density Estimates Across Time				
Neg-Binomial(Densities) ~ $\beta_0 + \beta_1$(Year)	3	-29.67	67.18	0.00
Poisson(Densities) ~ $\beta_0 + \beta_1$(Year)	2	-62.72	130.40	63.22
No. Density Estimates Across Time and Among Camera Placements				
Neg-Binomial(Densities) ~ $\beta_0 + \beta_1$(Year) + β_2(Camera) + β_3(Year × Camera)	7	-37.73	92.31	0.00
Poisson(Densities) ~ $\beta_0 + \beta_1$(Year) + β_2(Camera) + β_3(Year × Camera)	6	-89.06	192.20	99.89
No. Density Estimates Across Time and Among Velocity Sources				
Neg-Binomial(Densities) ~ $\beta_0 + \beta_1$(Year) + β_2(Velocity) + β_3(Year × Velocity)	5	-37.84	88.06	0.00
Poisson(Densities) ~ $\beta_0 + \beta_1$(Year) + β_2(Velocity) + β_3(Year × Velocity)	4	-81.79	173.14	85.08

[a] Model degrees of freedom.

[b] Log-likelihood of model.

[c] Difference between AIC_c of model and AIC_c of top-ranked model.
