# Supplementary figures and images for: Most Random-Encounter-Model Density Estimates in Camera-Based Predator–Prey Studies Are Unreliable
Source: Animals (Basel). 2024 Nov 22;14(23):3361. doi: 10.3390/ani14233361 (PMC11639839; doi:10.3390/ani14233361)

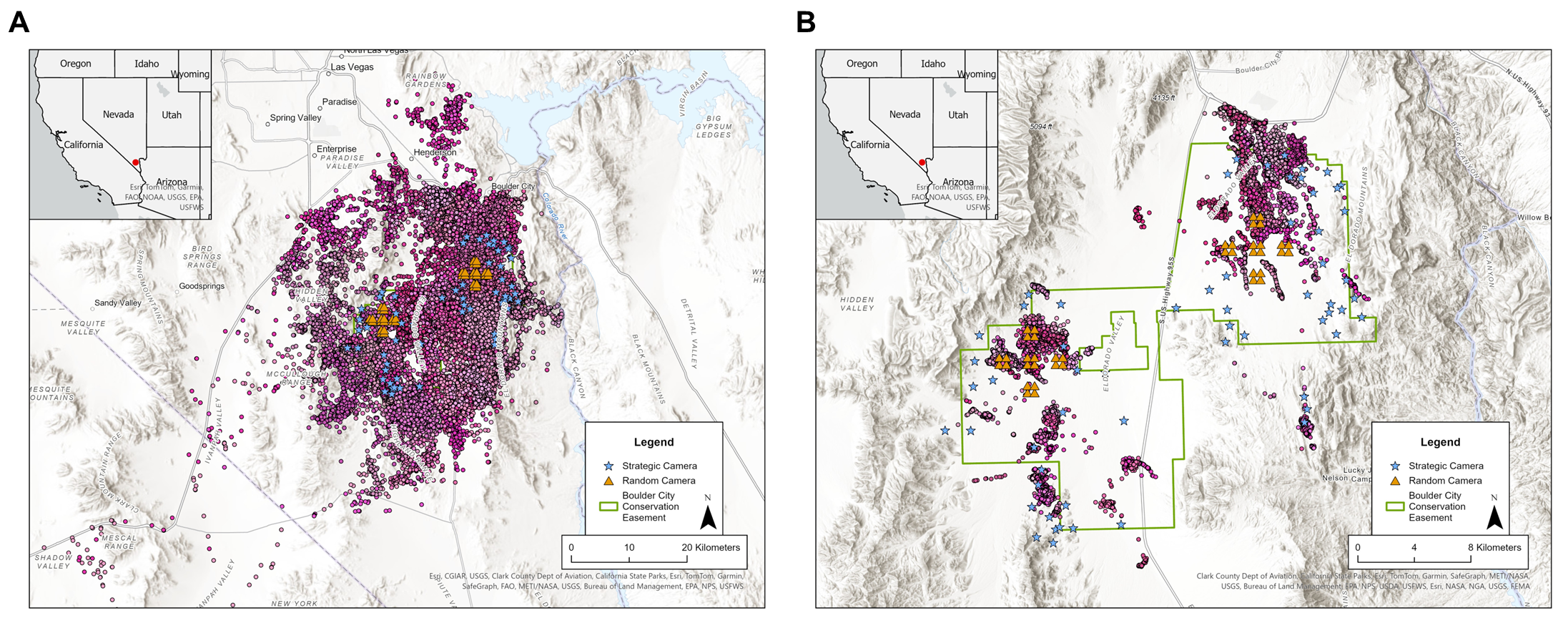

Supplement: Supplementary file 1 [file animals-14-03361-s001.zip › Figure S1.tif]
